# Decreased 5-hydroxymethylcytosine levels correlate with cancer progression and poor survival: a systematic review and meta-analysis

**DOI:** 10.18632/oncotarget.13719

**Published:** 2016-11-30

**Authors:** Zhaoli Chen, Xuejiao Shi, Lanwei Guo, Yuan Li, Mei Luo, Jie He

**Affiliations:** ^1^ Department of Thoracic Surgery, National Cancer Center/Cancer Hospital, Chinese Academy of Medical Sciences, Beijing, China; ^2^ Department of Cancer Epidemiology, Affiliated Cancer Hospital of Zhengzhou University, Henan Cancer Hospital, Henan Office for Cancer Control and Research, Zhengzhou, China

**Keywords:** 5-hydroxymethylcytosine, cancer staging, overall survival, disease-free survival, meta-analysis

## Abstract

Ten-eleven translocation (TET) enzymes catalyze the oxidation of 5-methylcytosine (5-mC) to 5-hydroxymethylcytosine (5-hmC) and then to 5-formylcytosine (5-fC) and 5-carboxylcytosine (5-caC), resulting in genomic DNA demethylation. Decreased 5-hmC levels have been reported in a variety of cancers, and loss of 5-hmC might be considered an epigenetic hallmark of cancer. However, the prognostic value of decreased 5-hmC in cancers remain controversial. Here, a systematic review was performed by conducting an electronic search of PubMed, EMBASE, Web of Science and the Cochrane Library. Finally, ten studies with a total of 1736 patients with cancer were included in the present study. Negative/low 5-hmC levels were significantly associated with lymph node metastasis [OR=2.20, 95% CI=1.23-3.96, *P*=0.008] and advanced TNM stage [OR=2.89, 95% CI=1.21-6.92, *P*=0.017]. More importantly, negative/low 5-hmC levels were significantly associated with poor prognosis of cancer patients [overall survival: HR=1.76, 95% CI=1.41-2.11, *P* < 0.001; disease free survival: HR=1.28, 95% CI=0.60-1.96, *P* < 0.001]. The results of this meta-analysis indicate that decreased 5-hmC levels are an indicator of poor survival of cancer patients. Given variability related to ethnicity, cancer types and detection methods, additional well-designed studies with larger sample sizes are required to further confirm our findings.

## INTRODUCTION

Cancer is characterized by genetic and epigenetic aberrations. DNA methylation, an important epigenetic mark that is dysregulated in many cancers, predominantly occurs on the C-5 atom of cytosine in the context of CpG islands in mammals (5-mC) and modulates gene activity by interacting with methyl-binding proteins [1]. TET enzymes, a family of Fe(II)- and α-ketoglutarate (α-KG)-dependent dioxygenases, convert 5-mC to 5-hmC, which can be further converted to 5-fC and 5-caC by the same enzymes [2–4]. The generation of 5-hmC induces the reversal of the gene silencing effect of 5-mC because 5-hmC is not recognized by transcriptional repressors such as methyl-CpG binding domain proteins (MBD) [5, 6].

Penn et al. first discovered 5-hmC in the 1970s, and 5-hmC has since been detected in a variety of tumor tissues [7–9]. 5-hmC levels are decreased in various malignancies, including glioblastoma, melanoma, breast, prostate, hepatic, gastric and renal cancers [10–16]. Lian et al. observed that the loss of 5-hmC is an epigenetic hallmark of melanoma and that decreased 5-hmC levels are associated with poorer melanoma outcome [10]. Liu et al. observed that 5-hmC levels are decreased in hepatic cancer and that low 5-hmC is an independent prognostic indicator of both overall survival and time to recurrence [14]. Several recent studies have demonstrated that low 5-hmC expression is associated with poor prognosis in renal cell carcinoma, gastric and ovarian cancers [13, 15, 17]. However, controversial results have been reported. Strand et al. reported that decreased 5-hmC in prostate cancer was associated with improved biological disease-free survival [12]. Hence, we performed a systematic review of the current evidence using a meta-analysis to analyze the association between 5-hmC levels and the clinicopathological characteristics in several types of cancers and to investigate the value of 5-hmC in the prognosis indication of cancer patients.

## RESULTS

### Study characteristics

**Figure 1:**
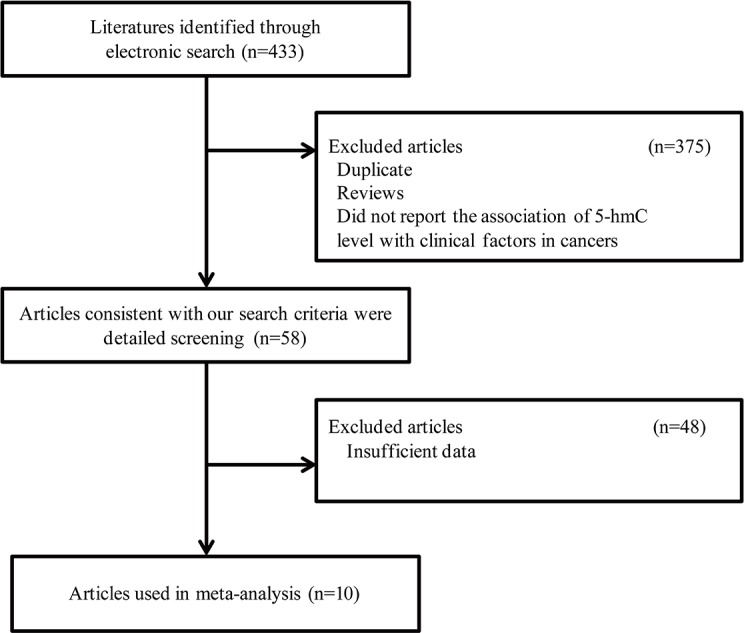
Flow diagram of the selection process

We initially identified four hundred thirty-three potentially relevant studies, and after reviewing the titles and abstracts, fifty-eight articles consistent with our search criteria were chosen for further screening. After careful evaluation, forty-eight studies with insufficient data were excluded. Ultimately, ten studies were chosen for the final analysis (Figure 1). The articles included one hepatocellular carcinoma study, one glioblastoma study, one gastric cancer study, one esophageal squamous cell carcinoma study, one prostate cancer study, one ovarian cancer study, one intrahepatic cholangiocarcinoma study, one kidney cancer study, one breast cancer study and one cervical cancer study [11, 12, 14–21]. The characteristics of the included studies are listed in Table 1.

**Table 1:** The characteristics of the included studies

Author	Year	Country	Cancer type	Patients' number	Detection methods	Cut-off for 5-hmC low expression
Orr	2012	America	Glioblastoma	69	IHC	H-score in the first quartile
Yang	2013	China	GC	108	DNA dot blot	< median value
Liu	2014	China	HCC	318	IHC	< median value
Murata	2015	Japan	ESCC	95	ELISA	< median value
Strand	2015	Denmark	PC	311	IHC	Scores 0 and 1 were considered as low expression
Zhang	2015	China	EOC	130	IHC	− and + were considered as low expression
Dong	2015	China	ICC	123	IHC	NR
Chen	2015	China	Kidney cancer	185	IHC	≤10% cells are positive for IHC staining
Tsai	2015	China	Breast cancer	257	IHC	The score range from 0-7, the cutoff set at a value of 3
Zhang	2016	China	CSCC	140	IHC	− and + were considered as low expression

Abbreviations: HCC, hepatocellular carcinoma; GC, gastric cancer; ESCC, esophageal squamous cell carcinoma; PC, prostate cancer; EOC, epithelial ovarian cancer; ICC, intrahepatic cholangiocarcinoma; CSCC, cervical squamous cell carcinoma; NR, not reported; IHC, immunohistochemistry; ELISA, enzyme-linked immunosorbent assay

### Meta-analysis results

Data relating to lymph node metastasis, TNM stage, histology grade and survival were extracted and are presented in Tables 2 and 3.

**Table 2:** The clinicopathological characteristics of the cases with positive/high 5-hmC and cases with the negative/low 5-hmC in tumor tissues

Study	Year	Cases (positive/high 5-hmC)							Cases (negative/low 5-hmC)						
		Total	N0	N1	I/II	III/IV	G1/2	G3/4	Total	N0	N1	I/II	III/IV	G1/2	G3/4
Yang	2013	50	27	23	30	20	25	25	58	15	43	13	45	27	31
Liu	2014	159	NR	NR	NR	NR	115	44	159	NR	NR	NR	NR	129	30
Murata	2015	48/46	14	34	19	27	43	5	47/46	16	31	20	26	32	15
Zhang	2015	34	18	16	23	11	30	4	96	29	67	34	62	67	29
Dong	2015	20	19	1	18	2	9	11	103	74	29	68	35	52	51
Zhang	2016	43	32	11	NR	NR	35	8	97	55	42	NR	NR	56	41

**Table 3:** The HR value and 95%CI of OS and DFS in each study

Study	Year	OS		DFS		Survival results
		HR	95%CI	HR	95%CI	
Orr	2012	2.28	1.16-4.49	NA	NA	poor
Yang	2013	2.55	1.30-4.98	NA	NA	poor
Liu	2014	3.16	2.12-4.74	NA	NA	poor
Chen	2015	2.22	1.19-4.17	NA	NA	poor
Dong	2015	1.45	1.08-1.93	1.36	1.02-1.81	poor
Strand	2015	NA	NA	0.62	0.44-0.87	well
Tsai	2015	NA	NA	1.90	1.20-3.02	poor
Zhang	2015	2.03	1.22-3.46	NA	NA	poor
Zhang	2016	2.02	0.93-7.16	2.56	1.06-6.24	poor

**Figure 2:**
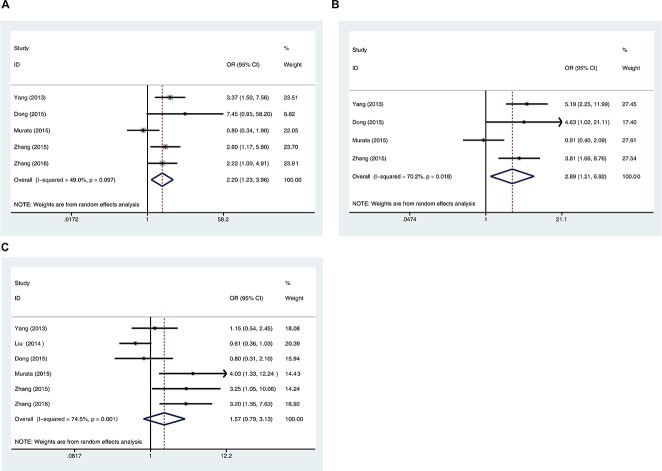
Correlation of 5-hmC levels and cancer stages **A.** Forest plots of the association between negative/low 5-hmC levels and lymph node metastasis (positive vs negative). **B.** Forest plots of the association between negative/low 5-hmC levels and TNM stage (III/IV stage vs I/II stage). **C.** Forest plots of the association between negative/low 5-hmC levels and histological differentiation grade (poor vs well/moderate). The squares and horizontal lines correspond to the study-specific OR and 95% CI, respectively. The area of the squares reflects the weight (inverse of the variance). The diamond represents the summary OR and 95% CI.

Five studies that collectively including 596 cases were used to evaluate the relationship between 5-hmC levels and lymph node metastasis. Meta-analysis using the random-effect model revealed that the negative/low expression rate of 5-hmC was higher in cancer patients with positive LN metastasis than in those with negative LN metastasis. The association of negative/low 5-hmC level and positive lymph node metastasis was statistically significant in different cancers [OR = 2.20, 95% CI = 1.23-3.96, *P* = 0.008] (Figure 2A).

Four studies collectively including 453 cases were used to evaluate the relationship between the 5-hmC level and TNM stage of cancer. Meta-analysis using the random-effect model demonstrated that the negative/low expression rate of 5-hmC was higher in cancer patients with TNM III/IV stage than in those with I/II stage. The association of negative/low 5-hmC levels and advanced TNM stage was statistically significant in different cancers [OR = 2.89, 95% CI = 1.21-6.92, *P* = 0.017] (Figure 2B).

Six studies collectively including 914 cases were used to evaluate the relationship between the 5-hmC level and histology grade of cancer. These six studies reported decreased 5-hmC in the poor differentiation group compared to the well-moderate differentiation group; however, meta-analysis using the random-effect model revealed no significant main effect [OR = 1.57, 95% CI = 0.79-3.13, *P* = 0.195] (Figure 2C).

**Figure 3:**
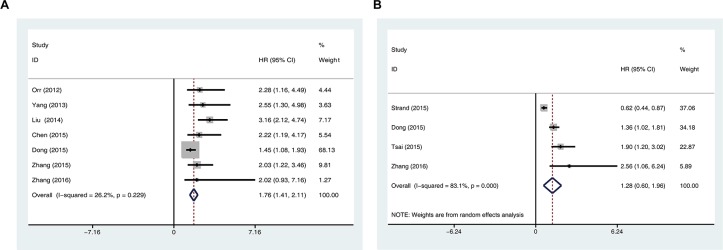
Prognostic value of negative/low 5-hmC levels in patients with cancer **A.** Meta-analysis of the association between negative/low 5-hmC levels and overall survival in various cancers. **B.** Meta-analysis of the association between negative/low 5-hmC levels and disease-free survival in various cancers.

Seven studies reported overall survival (OS) of 1073 cancer patients, and four studies reported the disease-free survival (DFS) of 831 cancer patients according to 5-hmC level in tumor tissues. The fixed-effect model and random-effect model were used to calculate the pooled HR with corresponding 95% CI for OS and DFS, respectively. The meta-analysis results showed that negative/low 5-hmC levels were significantly associated with poor survival of patients with various cancers [OS: HR = 1.76, 95% CI = 1.41-2.11, *P* < 0.001; DFS: HR = 1.28, 95% CI = 0.60-1.96, *P* < 0.001] (Figure 3). These results suggest that negative/low 5-hmC levels might be an indicator of poor prognosis for cancer patients.

### Tests of heterogeneity

**Figure 4:**
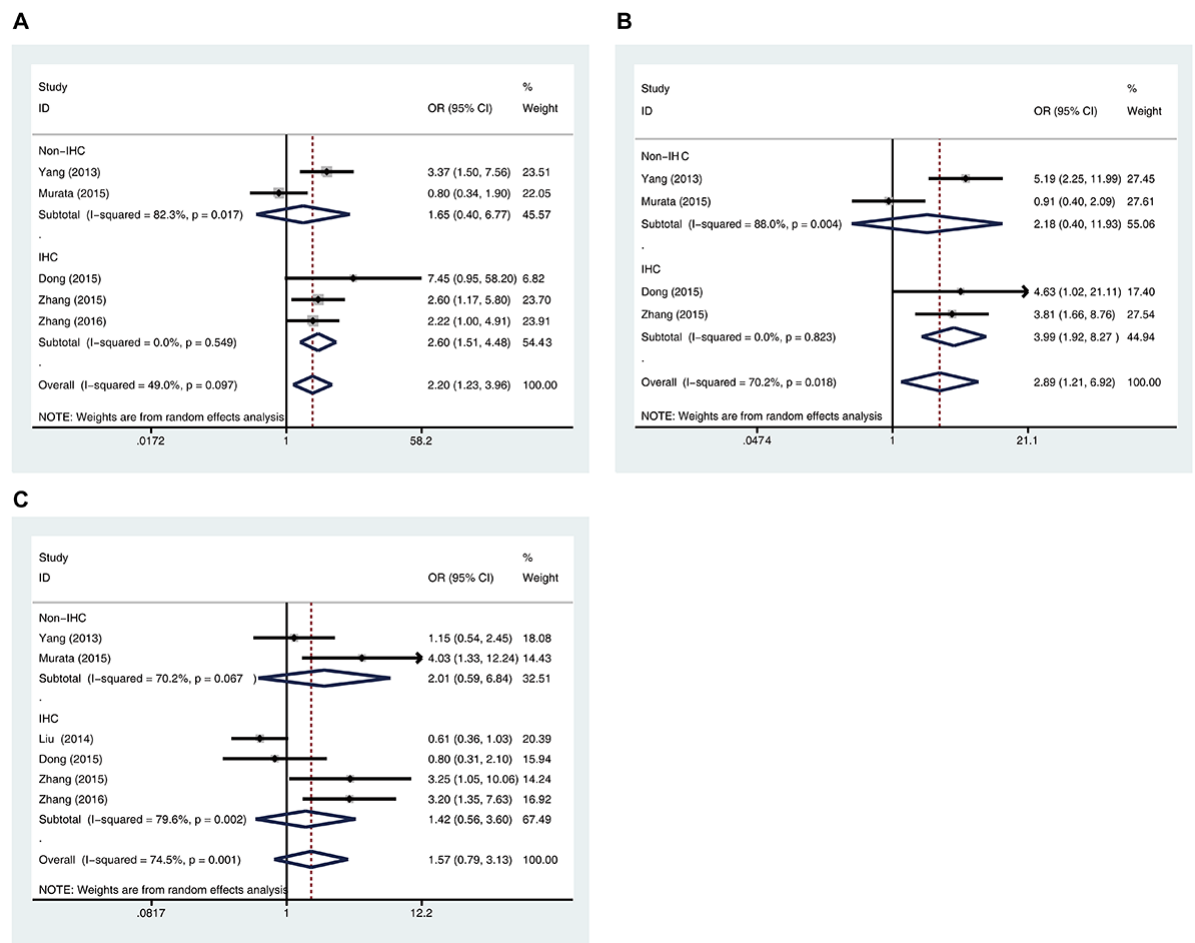
Stratified analyses of the association between 5-hmC levels and lymph node metastasis A., TNM stage B., and histological differentiation grade C. were performed by detection methods

The inter-study heterogeneity among the included studies were assessed using the *Q* test. Moderate to high heterogeneity was detected in the data on the association of negative/low 5-hmC with positive lymph node metastasis (*P* = 0.097, *I^2^* = 49.0%), advanced TNM stage (*P* = 0.018, *I^2^* = 70.2%), poor pathological differentiation (*P* = 0.001, *I^2^* = 74.5%), and poor DFS (*P* < 0.001, *I^2^* = 83.1%); thus, the random-effects models were used for these analyses. There was no obvious heterogeneity in the data on the association of 5-hmC with OS (*P* = 0.229, *I^2^* = 26.2%); thus, the analysis was performed using a fixed-effects model. To explore the source of heterogeneity, stratified analyses by detection method were performed (Figure 4), which revealed that heterogeneity could be reduced in all analyses except histology grade.

### Publication bias

**Figure 5:**
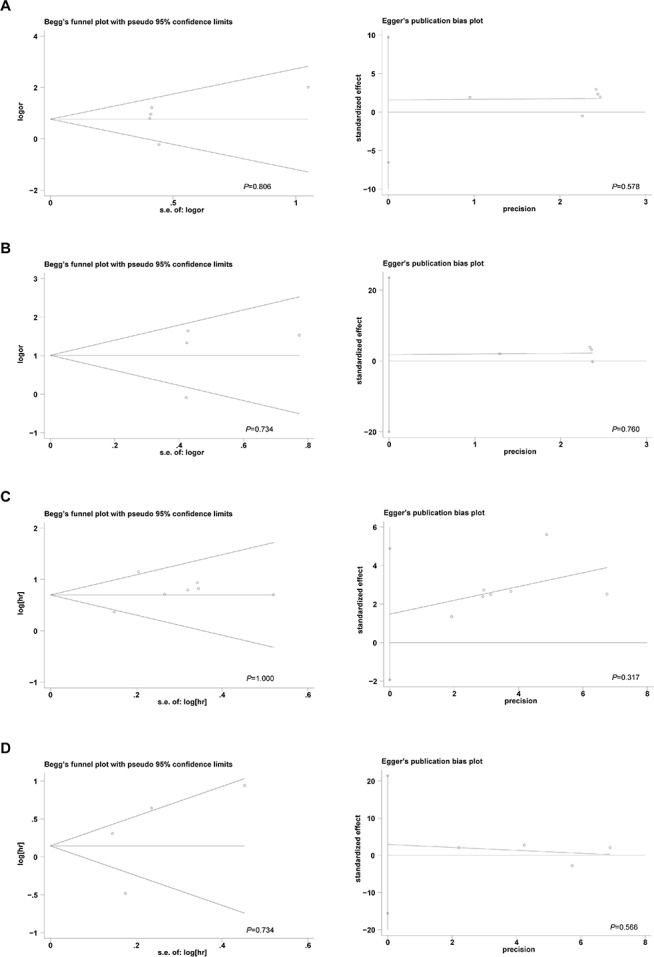
The Begg's funnel plots and Egger's publication bias plots assessing the publication bias in analyses of the association of 5-hmC levels with lymph node metastasis A., TNM stage B., overall survival C., and disease-free survival D

We assessed potential publication bias statistically using Begg's and Egger's tests (Figure 5). The funnel plots were symmetrical, and the results of the Begg's and Egger's tests revealed no publication bias (Begg's test, *P* > 0.05; Egger's test, *P* > 0.05).

## DISCUSSION

DNA methylation is one of the most-studied epigenetic modifications and plays an important role in carcinogenesis [22]. DNA methylation occurs predominately on the C-5 atom of cytosine in the context of CpG islands in mammals (5-mC) and is associated with the alteration of chromatin organization and modulation of gene activity [23]. 5-mC can be converted to 5-hmC by TET family enzymes [3], and the generation of 5-hmC modulates 5-mC-dependent gene regulation. Thus, 5-hmC might be a potential diagnostic, prognostic and predictive marker for cancers [24–26]. Ha et al. observed that immuno-assessment of 5-hmC could diagnostically differentiate benign and malignant melanocytic neoplasms; 88% of nevi were positive for 5-hmC, whereas only 31% of melanomas were 5-hmC positive. Moreover, nodular, lentigo maligna and desmoplastic melanoma did not exhibit positive 5-hmC, whereas 47% of the remaining melanoma subtypes and metastases melanoma were 5-hmC positive [27]. Liu et al. reported that 5-hmC correlated with less aggressive tumor behavior in hepatic cancer, and low 5-hmC levels were associated with poor survival and could serve as a prognostic marker for HCC [14]. However, the relationship between 5-hmC levels and survival in other cancers is still not completely understood. Moreover, the clinical significance of 5-hmC in various cancers remains controversial. To our knowledge, the present study is the first to evaluate the correlation between 5-hmC levels and the prognosis of various cancers. Our meta-analysis provides evidence of a significant association between negative/low 5-hmC levels and lymph node metastasis and advanced TNM stage in different cancers. More importantly, negative/low 5-hmC levels were significantly associated with poorer OS and DFS of cancer patients, suggesting that 5-hmC might be a prognostic marker for cancers.

Decreased 5-hmC levels in malignant tumors could be due to the mutation of TET genes or the mutations of isocitrate dehydrogenase 1 or 2 (IDH1/2), as mutation of IDH1/2 produces 2-hydroxyglutarate (2-HG) to inhibit the activity of TET proteins [28, 29]. Moreover, other studies have suggested that decreased 5-hmC levels might be caused by decreased TET1/2/3 expression [30, 31]. Decreased 5-hmC levels can be detected by various methods, including IHC, DNA dot blot, ELISA, and mass spectrometry. In our study, two papers included data from non-IHC detection that revealed high heterogeneity in the comparison of negative/low 5-hmC and lymph node metastasis and TNM stage. However, the high heterogeneity in the comparison of the association of negative/low 5-hmC levels with pathological differentiation grade was not significantly decreased by stratified analyses, suggesting that other factors, such as cancer type and varying cutoff values may be involved in the heterogeneity, which need to be further confirmed in future studies.

Although this systematic review aimed to provide a comprehensive estimate of the clinical significance of altered 5-hmC levels in various types of cancer, several limitations remain. First, the numbers of studies and patients included in this analysis were relatively small. Second, most of the studies were based on Asian populations, and only two studies analyzed the data in Caucasian populations. Third, low levels and loss of 5-hmC expression were reported together, preventing the evaluation of the difference between the associations of low and loss of 5-hmC expression with clinical parameters of cancer. Additional studies are needed to confirm these results.

In conclusion, our results indicate that advanced TNM stage and positive lymph node metastasis are more frequent in patients with negative/low levels of 5-hmC. Furthermore, we provide evidence for an association of negative/low 5-hmC with poor OS and DFS in various cancers. Therefore, decreased 5-hmC appears to be a valuable prognostic factor for cancers.

## MATERIALS AND METHODS

### Publication search strategy

A comprehensive literature search of PubMed, EMBASE, Web of Science and the Cochrane Library was performed up to March 2016. Search terms included “5-hydroxymethylcytosine OR 5-hmC OR 5hmC OR 5 hmC” combined with “neoplasm OR neoplasia OR tumor OR cancer.” Additional relevant references cited in the retrieved articles were also evaluated.

### Selection criteria

To be eligible for inclusion in the systematic review, a study was required to meet the following criteria: (1) detected 5-hmC levels in tumor tissues from any type of solid tumor, rather than in serum, cell lines or any other type of specimen; (2) published in English with full text available; (3) compared the difference between at least two groups (for example, positive/high 5-hmC compared to negative/low 5-hmC); (4) hazard ratios for OS or DFS with 95% CIs were available. Based on these criteria, ten eligible studies were included in this meta-analysis.

### Data collection

Publication characteristic details, such as the first author's name, publication year, patients' country, the number of patients, cancer type, detection methods, clinicopathological features, the cut-off definition of low 5-hmC and the HR for OS and/or DFS with corresponding 95% CIs were collected. When both univariate analysis and multivariate analysis were reported, the results of the multivariate analysis were used for analysis.

### Statistical analysis

All statistical analyses were performed using the STATA 12.0 software package (Stata Corporation, College Station, TX, USA). The associations between 5-hmC levels and lymph node metastasis, TNM stage and histology grade were evaluated by calculating the ORs and corresponding 95% CIs. For survival data, the HRs with 95% CIs were extracted, and pooled HRs and corresponding 95% CIs were calculated. The Cochrane *Q* test *(P* < 0.10 indicated a high level of statistical heterogeneity) and *I*^2^ (values of 25%, 50% and 75% corresponding to low, moderate and high degrees of heterogeneity, respectively) were used to assess the heterogeneity among eligible studies [32]. When no statistically significant heterogeneity was observed, a pooled effect was calculated using a fixed-effects model; otherwise, a random-effects model was used. Publication bias was evaluated using Begg's and Egger's tests. All *P* values in the meta-analysis were two-sided, and a *P* value of less than 0.05 was considered significant.

## References

[1] Baylin SB, Jones PA (2011). A decade of exploring the cancer epigenome - biological and translational implications. Nat Rev Cancer.

[2] Tahiliani M, Koh KP, Shen YH, Pastor WA, Bandukwala H, Brudno Y, Agarwal S, Iyer LM, Liu DR, Aravind L, Rao A (2009). Conversion of 5-methylcytosine to 5-hydroxymethylcytosine in mammalian DNA by MLL partner TET1. Science.

[3] Ito S, Shen L, Dai Q, Wu SC, Collins LB, Swenberg JA, He C, Zhang Y (2011). Tet Proteins can convert 5-methylcytosine to 5-formylcytosine and 5-carboxylcytosine. Science.

[4] He YF, Li BZ, Li Z, Liu P, Wang Y, Tang QY, Ding JP, Jia YY, Chen ZC, Li L, Sun Y, Li XX, Dai Q (2011). Tet-mediated formation of 5-carboxylcytosine and its excision by TDG in mammalian DNA. Science.

[5] Jin SG, Kadam S, Pfeifer GP (2010). Examination of the specificity of DNA methylation profiling techniques towards 5-methylcytosine and 5-hydroxymethylcytosine. Nucleic Acids Res.

[6] Hashimoto H, Liu Y, Upadhyay AK, Chang Y, Howerton SB, Vertino PM, Zhang X, Cheng X (2012). Recognition and potential mechanisms for replication and erasure of cytosine hydroxymethylation. Nucleic Acids Res.

[7] Penn NW, Suwalski R, O'Riley C, Bojanowski K, Yura R (1972). The presence of 5-hydroxymethylcytosine in animal deoxyribonucleic acid. Biochem J.

[8] Kriaucionis S, Heintz N (2009). The nuclear DNA base 5-hydroxymethylcytosine is present in Purkinje neurons and the brain. Science.

[9] Haffner MC, Chaux A, Meeker AK, Esopi DM, Gerber J, Pellakuru LG, Toubaji A, Argani P, Iacobuzio-Donahue C, Nelson WG, Netto GJ, De Marzo AM, Yegnasubramanian S (2011). Global 5-hydroxymethylcytosine content is significantly reduced in tissue stem/progenitor cell compartments and in human cancers. Oncotarget.

[10] Lian CG, Xu Y, Ceol C, Wu F, Larson A, Dresser K, Xu W, Tan L, Hu Y, Zhan Q, Lee CW, Hu D, Lian BQ (2012). Loss of 5-hydroxymethylcytosine is an epigenetic hallmark of melanoma. Cell.

[11] Tsai KW, Li GC, Chen CH, Yeh MH, Huang JS, Tseng HH, Fu TY, Liou HH, Pan HW, Huang SF, Chen CC, Chang HY, Ger LP (2015). Reduction of global 5-hydroxymethylcytosine is a poor prognostic factor in breast cancer patients, especially for an ER/PR-negative subtype. Breast Cancer Res Treat.

[12] Strand SH, Hoyer S, Lynnerup AS, Haldrup C, Storebjerg TM, Borre M, Orntoft TF, Sorensen KD (2015). High levels of 5-hydroxymethylcytosine (5hmC) is an adverse predictor of biochemical recurrence after prostatectomy in ERG-negative prostate cancer. Clin Epigenetics.

[13] Du C, Kurabe N, Matsushima Y, Suzuki M, Kahyo T, Ohnishi I, Tanioka F, Tajima S, Goto M, Yamada H, Tao H, Shinmura K, Konno H (2015). Robust quantitative assessments of cytosine modifications and changes in the expressions of related enzymes in gastric cancer. Gastric Cancer.

[14] Liu WR, Tian MX, Jin L, Yang LX, Ding ZB, Shen YH, Peng YF, Zhou J, Qiu SJ, Dai Z, Fan J, Shi YH (2014). High expression of 5-hydroxymethylcytosine and isocitrate dehydrogenase 2 is associated with favorable prognosis after curative resection of hepatocellular carcinoma. J Exp Clin Cancer Res.

[15] Chen K, Zhang J, Guo Z, Ma Q, Xu Z, Zhou Y, Xu Z, Li Z, Liu Y, Ye X, Li X, Yuan B, Ke Y (2015). Loss of 5-hydroxymethylcytosine is linked to gene body hypermethylation in kidney cancer. Cell Res.

[16] Orr B, Haffner M, Eberhart C, Hicks J, Nelson W, Yegnasubramanian S (2012). Decreased 5HMC is associated with neural progenitor phenotype in normal brain and shorter survival in malignant glioma. J Neuropathol Exp Neurol.

[17] Zhang LY, Li PL, Wang TZ, Zhang XC (2015). Prognostic values of 5-hmC, 5-mC and TET2 in epithelial ovarian cancer. Arch Gynecol Obstet.

[18] Murata A, Baba Y, Ishimoto T, Miyake K, Kosumi K, Harada K, Kurashige J, Iwagami S, Sakamoto Y, Miyamoto Y, Yoshida N, Yamamoto M, Oda S (2015). TET family proteins and 5-hydroxymethylcytosine in esophageal squamous cell carcinoma. Oncotarget.

[19] Dong ZR, Zhang C, Cai JB, Zhang PF, Shi GM, Gao DM, Sun HC, Qiu SJ, Zhou J, Ke AW, Fan J (2014). Role of 5-hydroxymethylcytosine level in diagnosis and prognosis prediction of intrahepatic cholangiocarcinoma. Tumor Biol.

[20] Yang Q, Wu K, Ji M, Jin W, He N, Shi B, Hou P (2013). Decreased 5-hydroxymethylcytosine (5-hmC) is an independent poor prognostic factor in gastric cancer patients. J Biomed Nanotechnol.

[21] Zhang LY, Han CS, Li PL, Zhang XC (2016). 5-Hydroxymethylcytosine expression is associated with poor survival in cervical squamous cell carcinoma. Jpn J Clin Oncol.

[22] Esteller M (2008). Epigenetics in cancer. N Engl J Med.

[23] Tan L, Shi YG (2012). Tet family proteins and 5-hydroxymethylcytosine in development and disease. Development.

[24] Kriukiene E, Liutkeviciute Z, Klimasauskas S (2012). 5-Hydroxymethylcytosine—the elusive epigenetic mark in mammalian DNA. Chem Soc Rev.

[25] Laird A, Thomson JP, Harrison DJ, Meehan RR (2013). 5-hydroxymethylcytosine profiling as an indicator of cellular state. Epigenomics.

[26] Vasanthakumar A, Godley LA (2015). 5-hydroxymethylcytosine in cancer: significance in diagnosis and therapy. Cancer Genet.

[27] Ha TT, Wang M, Hamarsheh S, Andea AA (2014). 5-hydroxymethylcytosine (5HMC) as an ancillary aid in the diagnosis of melanocytic lesions. Lab Invest.

[28] Dang L, White DW, Gross S, Bennett BD, Bittinger MA, Driggers EM, Fantin VR, Jang HG, Jin S, Keenan MC, Marks KM, Prins RM, Ward PS (2009). Cancer-associated IDH1 mutations produce 2-hydroxyglutarate. Nature.

[29] Delhommeau F, Dupont S, Della Valle V, James C, Trannoy S, Masse A, Kosmider O, Le Couedic JP, Robert F, Alberdi A, Lecluse Y, Plo I, Dreyfus FJ (2009). Mutation in TET2 in myeloid cancers. N Engl J Med.

[30] Liu C, Liu L, Chen X, Shen J, Shan J, Xu Y, Yang Z, Wu L, Xia F, Bie P, Cui Y, Bian XW, Qian C (2013). Decrease of 5-hydroxymethylcytosine is associated with progression of hepatocellular carcinoma through downregulation of TET1. PLoS One.

[31] Yang H, Liu Y, Bai F, Zhang JY, Ma SH, Liu J, Xu ZD, Zhu HG, Ling ZQ, Ye D, Guan KL, Xiong Y (2013). Tumor development is associated with decrease of TET gene expression and 5-methylcytosine hydroxylation. Oncogene.

[32] Higgins JP, Thompson SG (2002). Quantifying heterogeneity in a meta-analysis. Stat Med.

